# Sequential Flash NanoPrecipitation for the scalable formulation of stable core-shell nanoparticles with core loadings up to 90%

**DOI:** 10.1016/j.ijpharm.2023.122985

**Published:** 2023-06-10

**Authors:** Nicholas J. Caggiano, Satya K. Nayagam, Leon Z. Wang, Brian K. Wilson, Parker Lewis, Shadman Jahangir, Rodney D. Priestley, Robert K. Prud'homme, Kurt D. Ristroph

**Affiliations:** a Department of Chemical and Biological Engineering, Princeton University, Princeton, NJ 08544, United States; b Princeton Materials Institute, Princeton University, Princeton, NJ 08544, United States

## Abstract

Flash NanoPrecipitation (FNP) is a scalable, single-step process that uses rapid mixing to prepare nanoparticles with a hydrophobic core and amphiphilic stabilizing shell. Because the two steps of particle self-assembly – (1) core nucleation and growth and (2) adsorption of a stabilizing polymer onto the growing core surface – occur simultaneously during FNP, nanoparticles formulated at core loadings above approximately 70% typically exhibit poor stability or do not form at all. Additionally, a fundamental limit on the concentration of total solids that can be introduced into the FNP process has been reported previously. These limits are believed to share a common mechanism: entrainment of the stabilizing polymer into the growing particle core, leading to destabilization and aggregation. Here, we demonstrate a variation of FNP which separates the nucleation and stabilization steps of particle formation into separate sequential mixers. This scheme allows the hydrophobic core to nucleate and grow in the first mixing chamber unimpeded by adsorption of the stabilizing polymer, which is later introduced to the growing nuclei in the second mixer. Using this Sequential Flash NanoPrecipitation (SNaP) technique, we formulate stable nanoparticles with up to 90% core loading by mass and at 6-fold higher total input solids concentrations than typically reported.

## Graphical abstract

**Figure:**
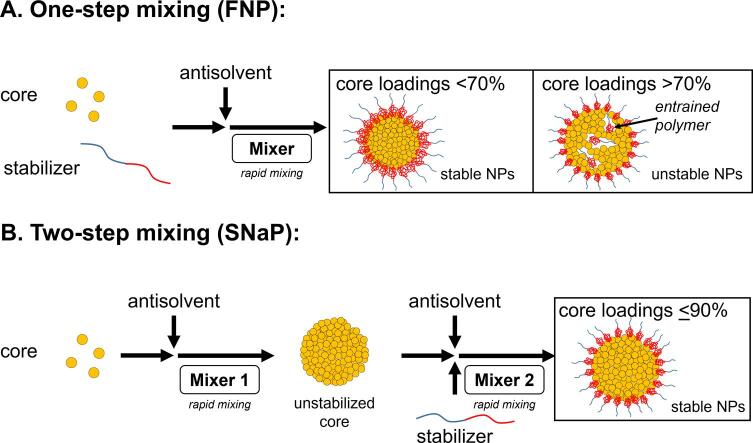

## 1 Introduction

Nanoparticle (NP) delivery of hydrophobic compounds is of increasing interest for solubility enhancement since a large proportion of new small molecule drugs exhibit poor solubility in water, thereby limiting oral bioavailability (Lipinski, 2000, Savjani et al., 2012, Thayer, 2010). NP delivery is also of interest for parenteral and oral delivery of hydrophilic compounds, including biomolecules such as proteins, peptides, and nucleic acids (Anselmo et al., 2019, Paunovska et al., 2022). Several approaches to prepare nanoparticles have been presented and reviewed;(Khan et al., 2019, Kumar et al., 2020) however, many techniques are limited by low core loading (mass fraction of core material per mass of total NP) or poor process scalability (Cai et al., 2015, Qin et al., 2017, Shen et al., 2017, Zhang et al., 2015). Our research has focused on a kinetically controlled, polymer-directed, rapid precipitation process termed Flash NanoPrecipitation (FNP). FNP relies on controlled turbulent micromixing in specially designed mixing chambers to produce supersaturations as high as 10,000 in 1.5 ms (Johnson and Prud’homme, 2003a, Pinkerton et al., 2015). The technique is scalable and has been demonstrated to produce the same monodisperse nanoparticles from laboratory scales (milligrams) to industry scales (kilograms) (Bobbala et al., 2018, Feng et al., 2019a, Li et al., 2014, Yu et al., 2021, Zeng et al., 2019). Although FNP typically produces NPs with core loadings of 33–70%, even higher drug loadings are desired to reduce the mass of excipients relative to the therapeutic compound (Saad and Prud’homme, 2016, Shen et al., 2017, Tao et al., 2019, Zhu, 2014). Additionally, producing NPs at higher mass concentrations reduces solvent waste and improves process mass intensity.

**Fig. 1:**
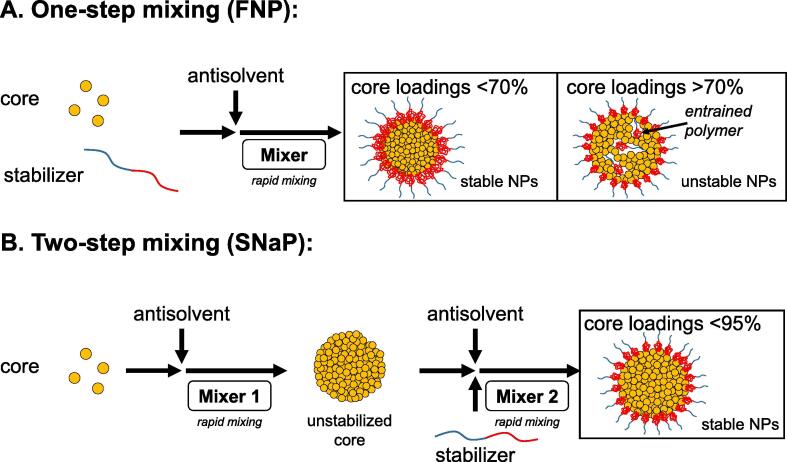
Principles of conventional FNP and sequential SNaP processes. (**A)** FNP utilizes a one-step mixing process where aggregation and stabilization occur simultaneously. (**B)** Proposed two-step mixing process where aggregation occurs followed by stabilization.

Rapid precipitation processes, including FNP, fail to produce stable nanoparticles when either (a) the total solids concentration during precipitation, or (b) the concentration of core material relative to the concentration of the stabilizer, is too high. Normally, the maximum achievable total solids concentration of NPs is approximately 0.1 wt% in the final dispersion (Pagels et al., 2018, Pustulka et al., 2013). At low total solids concentrations, the ratio of core material to stabilizer (e.g., drug loading) for nonionic hydrophobic species is usually limited to 20–50% (Pagels et al., 2018). The limitations in total solids and drug loading are related to the mechanism of self-assembly. It has been suggested that for formulations with core loadings above 70%, the ability of stabilizing polymer molecules to adsorb strongly onto a particle nucleus (so-called ‘anchoring efficiency’) is decreased due to the presence of stabilizer molecules which have become kinetically trapped within the growing particle core (Pustulka et al., 2013). This reduces the effective amount of available stabilizer and introduces packing inefficiencies in the drug-loaded NP core. The result is larger, more polydisperse nanoparticles which exhibit poor size stability over time (Fig. 1**A**).

Temporally separating the precipitation of the core materials from the adsorption of the stabilizing polymer surface material is a strategy to overcome the limitations of loading. One method to achieve this separation is through the use of mixed solvent systems to effect sequential separation of components. For example, Liu, Weitz, et al. demonstrated a two-step batch nanoprecipitation process where two sequential aliquots of antisolvent were added to an initial ternary solvent mixture containing core and polymer stabilizer. The first antisolvent addition caused precipitation of the core material, while the stabilizer remained soluble. After the second addition core–shell nanoparticles were produced with loadings of up to 59% (Han et al., 2020, Liu et al., 2020). A similar effect can also be produced in a continuous process using microfluidics to introduce antisolvents sequentially for the core and stabilizing compounds (Liu et al., 2017). However, both cases require careful design of solvent systems to achieve sequential precipitation. Additionally, there are limitations to scaling microfluidics to large-scale nanoparticle production. In microfluidics, rapid solvent diffusion is achieved using the micron length scales of the microfluidic device; in small geometries the flows are laminar. In contrast, during turbulent mixing in FNP the small length scales for diffusion are achieved by using turbulence to establish solvent-antisolvent striations at the Kolmogorov length scale (Johnson and Prud’homme, 2003b). FNP has been applied to various payloads and stabilizing polymer chemistries and is highly scalable (Feng et al., 2019a, Pinkerton et al., 2017, Pustulka et al., 2013, Yan et al., 2021, Yu et al., 2021, Zeng et al., 2019).

Our process, which we term *Sequential Flash NanoPrecipitation* (SNaP), is accomplished by the sequential arrangement of two mixing chambers. In the first chamber, the hydrophobic core material is precipitated by rapid mixing and antisolvent-induced supersaturation. Before the hydrophobic cores can aggregate beyond the desired size scale, they enter a second FNP mixing chamber where the stabilizing polymer is introduced, which adsorbs onto the NP core surfaces (Fig. 1**B**). Conceptually, this is similar to the ability to introduce coating layers onto nanoparticle surfaces via FNP, as we have demonstrated for the coating of latex spheres(D’Addio et al., 2012) or inorganic oxide colloids (Budijono et al., 2010, Ungun et al., 2009). In SNaP, the delay time between the initial core precipitation and the subsequent application of the stabilizing layer is determined by the residence time in the tubing connecting the first and second chambers. This delay time is intentionally kept to a minimum to limit the extent of core aggregation which occurs between the precipitation and coating steps.

## 2 Materials and methods

### 2.1 Reagents

Polymeric core material polycaprolactone diol (PCL, 2 kDa) was obtained from Sigma-Aldrich (St. Louis, MO) and used without further purification. Hydroxypropyl methylcellulose acetate succinate (HPMCAS; AQOAT® AS-HF) was generously provided by Shin-Etsu Chemical Co. (Tokyo, Japan). Tetrahydrofuran (THF, HPLC grade) was purchased from Fisher Chemical (Hampton, NH). Sodium hydroxide and sodium chloride were purchased from Sigma-Aldrich (St. Louis, MO). Deionized water (18.2 MΩ•cm) was prepared using a Thermo Scientific Nanopure Diamond water system (Waltham, MA).

### 2.2 Sequential mixing and nanoparticle characterization

#### 2.2.1 Preparation of FNP control formulations.

Flash NanoPrecipitation was performed using a confined impinging jet mixer (CIJ) as reported previously (Han et al., 2012, Markwalter et al., 2019, Saad and Prud’homme, 2016). In all nanoparticle formulations studied here, PCL was the core material and HPMCAS was the stabilizing material. In brief, a solvent stream was prepared by dissolving polycaprolactone (PCL, 2 kDa) in tetrahydrofuran at various concentrations. An aqueous antisolvent stream was prepared by dissolving HPMCAS free acid at various concentrations in water with 15 mM NaCl along with 1.08 equivalents of NaOH to ionize the succinyl groups on the polymer. The solvent and antisolvent solutions (0.5 mL each) were loaded into separate Luer-lip syringes, which were attached to a CIJ and rapidly depressed. The mixer effluent was immediately collected in a 4 mL water reservoir. Formulation details for conventional FNP formulations, including stream compositions, NP loadings, size, and polydispersity index can be found in Table S1.

#### 2.2.2 Preparation of SNaP formulations.

For sequential nanoprecipitation, two CIJ mixers were used. Two 2.5 mL glass Luer-lock syringes were attached to the first mixer, one containing 1 mL of a THF solvent stream with dissolved PCL and the other containing 1 mL of 15 mM NaCl in water. The effluent port from this mixer was attached to one inlet on the second CIJ mixer by PEEK tubing (described below). To the remaining port of the second mixer was attached a 5 mL glass Luer-lock syringe containing 2 mL of HPMCAS (with 1.08 equivalents of NaOH) at various concentrations in water with 7.5 mM NaCl. All three syringes were depressed simultaneously, and the effluent from the second mixer was immediately collected in a 6 mL water reservoir. Formulation details for SNaP formulations, including stream compositions, NP loadings, size, and polydispersity index can be found in Table S2.

#### 2.2.3 Size characterization by dynamic light scattering.

Following formulation, aliquots were removed from nanoparticle dispersions and diluted in water to a NP concentration of 0.1 mg mL^−1^ for characterization. Size and polydispersity index (PDI) were measured by dynamic light scattering (DLS) using a Zetasizer Nano-ZS (Malvern Instruments, Malvern, UK) (n = 3). Particle size is reported as Z-average hydrodynamic diameter obtained from intensity-weighted distributions. Nanoparticle stability was monitored and evaluated using both visual observation and DLS.

### 2.3 Equipment and mixing setups

A mixing stand was designed in-house to allow for depression of multiple syringes for the SNaP process in making small-scale formulations, as shown in Figure S1. The stand base plate was modified to seat two confined impinging jet (CIJ) mixers and, later, one CIJ and one multi-inlet vortex mixer (MIVM) (Liu et al., 2008, Markwalter et al., 2019). Detailed CAD drawings can be found in Figure S2. For SNaP, the outlet of the first mixer was connected to one of the inlets of the second mixer with 250 mm of 0.030-inch inner diameter polyether ether ketone (PEEK) tubing. Syringes were fixed on the remaining inlets of both mixers. The depression plate was then manually pushed down to initiate mixing. The time required to depress syringes using the mixing stand was measured as $t_{\mathrm{inject}}=0.35s$ using video footage of the process and averaging across multiple trials. This corresponds to a volumetric flow rate of 2.3 mL s^−1^. For SNaP, the total time between when a fluid element entered the mixing chamber of the first mixer (mixing chamber volume ∼ 0.2 mL) and the second mixer was 0.14 s.

## 3 Results and discussion

### 3.1 Mixing setup and rationale for sequential nanoprecipitation

**Fig. 2:**
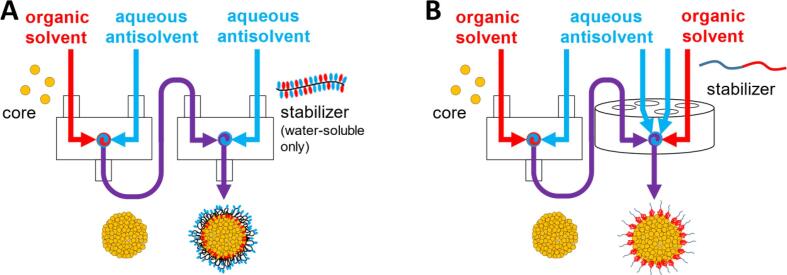
(**A)** Schematic of SNaP using two connected confined impinging jet (CIJ) mixers, or (**B)** a CIJ mixer and a multi-inlet vortex mixer. Core material is precipitated in the first mixer using an antisolvent stream before stabilizer is added in the second mixer.

The sequential mixing process can be divided into two steps: precipitation and stabilization (Fig. 2). Low molecular weight (2 kDa) PCL was used here as a model core material because it behaves similarly to nonionic, small-molecule hydrophobic drugs during the precipitation process. In the first mixing step, the nanoparticle core material (PCL) was dissolved in a water-miscible organic solvent (tetrahydrofuran) and then rapidly mixed against an aqueous antisolvent stream using a CIJ mixer. The antisolvent stream in FNP is typically deionized water; in this work, a NaCl concentration of 7.5 mM was maintained in all mixing chambers to prevent self-stabilization of polycaprolactone cores via the electrostatic surface charge that has been observed on hydrophobic surfaces in water (Roger and Cabane, 2012, Zhang et al., 2012). Control experiments performed with NaCl and without stabilizer showed that PCL did not self-stabilize and instead formed large aggregates.

Following the initial precipitation step, the effluent from the first CIJ mixer was fed *via* tubing to the second CIJ mixer, where it was mixed with a solution containing the nanoparticle surface stabilizing material. To avoid redissolution of the PCL cores formed in the first mixer, the solvent concentration in the second mixer must not be increased above that of the first mixer effluent (50% water / 50% THF, v/v). Since the CIJ mixer has only two inlets and requires nearly equivalent stream flow rates to drive momentum annihilation (Saad and Prud’homme, 2016), the two-CIJ-mixer setup in Fig. 2 requires that the solution introduced in the second mixer be primarily aqueous.

Due to this requirement, a water-soluble stabilizing material must be used in a sequential nanoprecipitation scheme using two CIJ mixers. In this work we used hydroxypropyl methylcellulose acetate succinate (HPMCAS), a semisynthetic cellulosic polymer reported previously as appropriately amphiphilic for application as a stabilizer in FNP nanoparticle formulations (Caggiano et al., 2022, Feng et al., 2019b, Feng et al., 2018, Ristroph et al., 2019). However, many of the polymers studied in the FNP literature are water-insoluble diblock copolymers that are not appropriate for this application. To use these in the SNaP process, the second mixer must have more than two inlets, or else not require equal stream volumes. The multi-inlet vortex mixer (MIVM)(Liu et al., 2008, Markwalter et al., 2019) with four inlets, which was developed for FNP as an alternative to the CIJ geometry, meets these requirements and will be a focus of future work on SNaP. Initial efforts to use an MIVM as the second mixer of SNaP (Fig. S1**D-E**) to enable a diblock copolymer stabilizer were attempted for a formulation with a vitamin E acetate core and poly(styrene)-b-poly(ethylene glycol) (PS-b-PEG) stabilizer at 83% core loadings. The resulting nanoparticles had a Z-average diameter of approx. 200 nm (Figure S3); however, further development for the CIJ-to-MIVM system will be reserved for future work.

### 3.2 Nanoparticle formation

#### 3.2.1 Governing variables for precipitation and self-assembly.

Pagels et al. showed that the size of NPs prepared by FNP is independent of the core material used, provided that material is sufficiently hydrophobic to rapidly precipitate in the mixed solvent environment of an FNP mixing chamber (log P_octanol/water_ > ∼4.5) (Pagels et al., 2018). Their work also showed that, for a given core material and given stabilizer, the two variables that control NP size are the core loading and total mass concentration (TMC) of the formulation. Core loading is discussed in the introduction: NP size increases as core loading increases, and NPs either rapidly destabilize or do not form altogether at core loadings above 75% for block copolymer stabilized nanoparticles.

Pagels defined the TMC in terms of the total solids in the organic feed stream to FNP (Pagels et al., 2018). Since in SNaP there are multiple feed streams and mixers, we here define the TMC as the sum of the mass of the core material and the mass of the stabilizer in the system divided by the final solution volume including the collection reservoir (in practice, the difference between this definition and that used in Pagels et al. is a factor of 10). Higher TMC during the formulation process yields a more concentrated final NP suspension, which translates to a reduction of post-processing times (e.g., spray drying times or ultrafiltration concentration times) and an improvement of the overall process mass intensity. For formulations of equal core loading, increasing the TMC increases the resulting NP size. Above a critical TMC, visible aggregates are observed, indicating that not all core material is encapsulated. As with the upper limit of core loading, the likely mechanism for this limitation is the entrainment of stabilizer molecules within NP cores. As TMC increases, the average distance between two adjacent molecules during NP assembly decreases, which can lead to stabilizer molecules becoming trapped among rapidly assembling core molecules. Typical TMCs for FNP formulations are around 1–2 mg mL^−1^ (Markwalter et al., 2019).

#### 3.2.2 Nanoparticle core loading.

**Fig. 3:**
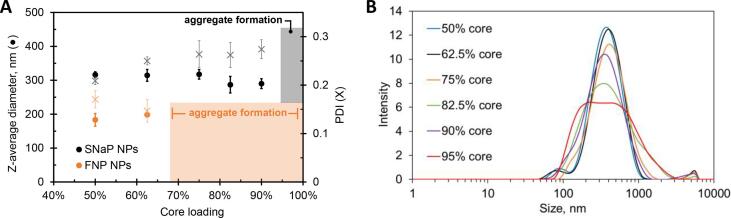
(A**)** Z-average size (circles) and polydispersity index (PDI, crosses) of NPs produced by SNaP (black) or conventional FNP (orange) plotted versus core loading for a fixed TMC of 1 mg mL^−1^, (**B)** DLS traces for the six SNaP formulations shown in (**A)**.

The average size and polydispersity index (PDI) of formulations prepared by FNP or SNaP at a fixed TMC of 1 mg mL^−1^ and different core loadings are given in Fig. 3**A**. Conventional FNP produced a combination of NPs and visible aggregates at core loadings of 75% and higher. In contrast, no visible aggregates were observed in SNaP formulations at core loadings of 50%, 63%, 75%, 83%, and 90%. At 95% core loading, some replicates exhibited aggregates while others did not. At 99% core loading aggregates were consistently observed. Therefore, the maximum achievable core loading for this system is likely in the range of 90–95%. It is important to note that DLS measurements did not always detect the aggregates, which tended to adhere to vial walls or form a film on the surface of the liquid in a vial. Close visual scrutiny was necessary to identify aggregates at this small scale (5–10 mL per formulation), and representative photographs are provided in the SI (Figure S4). DLS measurements for the conventional FNP formulations produced at loadings above 75% indicated that the population of non-aggregated NPs had smaller average diameter and lower PDI than corresponding SNaP NPs. However, these DLS measurements are not representative of the true particle size distribution inclusive of the population of aggregates. At pharmaceutical scale, the formation of aggregates would lead to product losses and reduced reproducibility, hindering scale-up efforts (Feng et al., 2019a, Li et al., 2014, Zeng et al., 2019). As such, DLS measurements for visibly aggregated NP formulations are not reported here.

As core loading was increased, the Z-average diameter of the SNaP NPs decreased slightly and the PDI increased slightly. Fig. 3**B** shows the intensity-weighted particle size distributions for the five SNaP formulations from Fig. 3**A**. All six DLS traces exhibited at least slight deviation from monomodality which became more prominent with increased core loading. This was reflected in increased PDI values.

#### 3.2.3 Total mass concentration during mixing.

**Fig. 4:**
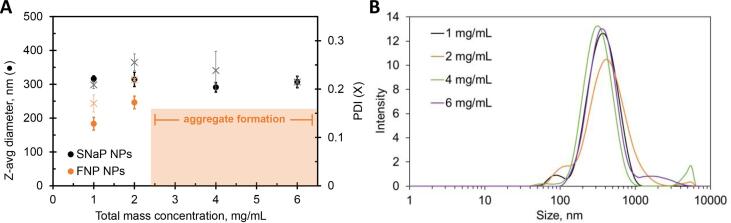
(A**)** Z-average size (circles) and polydispersity index (PDI, crosses) of NPs produced by SNaP (black) or conventional FNP (orange) plotted versus TMC for a fixed core loading of 50%, (**B)** DLS traces for the four SNaP formulations shown in (**A)**.

The average size and polydispersity index (PDI) of formulations prepared by FNP or SNaP at a fixed core loading of 50% and various TMCs are shown in Fig. 4. Above a TMC of 2 mg mL^−1^, conventional FNP produced aggregates along with NPs, whereas SNaP produced NPs without aggregates at TMCs up to 6 mg mL^−1^, representing a six-fold increase in the solids concentration in the effluent from the two-mixer setup. Again, close visual scrutiny was necessary to identify aggregates in conventional FNP formulations at this scale; DLS measurements alone were not sufficient to determine whether aggregation occurred (Figure S4).

#### 3.2.4 Particle size.

For formulations in which both methods yielded NPs without aggregates in Fig. 3, Fig. 4, NPs made by sequential precipitation were consistently larger and had higher PDI values than those made by single-step precipitation. This was in line with expectations, as NP cores in the sequential precipitation process had more time to aggregate before growth was arrested by HPMCAS adsorption in the second mixer. As described below, however, the delay time between mixers does not fully account for this difference in size.

Pagels modified Smoluchowski’s model for diffusion-limited aggregation to model NP assembly under FNP conditions (Pagels et al., 2018). One equation from the modified model can be used to back-calculate the amount of time required for NPs to assemble:

$$r={\left(\frac{tk_{B}Tc_{\mathrm{core}}}{\pi \mu \rho_{\mathrm{core}}}\right)}^{\frac{1}{3}}$$

where NP radius r (meters) may be written as a function of time t (seconds), absolute temperature T, concentration of core material $c_{\mathrm{core}}$ (kg m^−3^) solution viscosity μ (kg m^−1^ s^−1^), core material density $\rho_{\mathrm{core}}$ (kg m^−3^), and the Boltzmann constant $k_{B}$ (m^2^ kg s^−2^ K^−1^).

This model was used to calculate the difference in expected assembly time between the two comparable formulations at 62.5% core loading in Fig. 3. These calculations estimate that the 196 nm diameter particles produced by conventional FNP assembled over approximately 520 ms, compared with 2,700 ms for the 340 nm diameter SNaP NPs. As the delay time between the entrance from one mixer to the next mixers was estimated at only 140 ms, this delay alone does not appear to fully account for the 2,200 ms difference in predicted assembly time.

Importantly, the residence time in a CIJ under standard operation is only about 90 ms. For NP formulations with elevated core loadings or high TMCs, then, the model predicts that particle assembly is not complete by the time a fluid element exits the final mixer of either conventional FNP or SNaP. Here, the mixer effluent containing still-assembling NPs was collected in a downstream quench reservoir, where mixing was not as finely controlled as the rapid turbulent mixing inside the CIJ. This may have contributed to the larger sizes and higher PDI values of the final SNaP NPs. Despite the difference in final size (discussed further below), the ‘pre-templating’ of the SNaP cores appears to have enabled stable particle formation at high core loadings and TMCs without the formation of visible aggregates. The 140 ms of time for PCL cores to nucleate in SNaP prior to HPMCAS exposure in the second mixer should have been sufficient for nuclei 125 nm in diameter to form. These cores then began to be coated by HPMCAS in the second mixer.

If both formulations finished assembling after (poorly-controlled) dilution into additional water downstream of the good mixing – dilution which lowered the TMC and affected solvent quality (and therefore supersaturation) for this final stage of assembly – the NP cores made by conventional FNP may have been smaller because of longer exposure to the HPMCAS stabilizer. NP cores formed by conventional FNP were exposed to HPMCAS for all of the pre-quench assembly time, whereas cores formed by SNaP were only exposed to HPMCAS for less than half of the pre-quench assembly time.

The above example is an important point that should be considered when preparing NPs by FNP using a CIJ mixer. For NPs that are expected to take much longer than 90 ms to assemble (predictable as a function of TMC, core loading, and stabilizer), some or most particle assembly takes place downstream of the mixer. In industrial applications, the MIVM geometry removes the need for a downstream quench reservoir and is, therefore, recommended for formulations (whether SNaP or conventional FNP) made at high TMCs and/or high core loadings, which are expected to take more time to assemble than the residence time in the mixer.

#### 3.2.5 Nanoparticle size stability

All formulations made by SNaP in Fig. 3, Fig. 4 remained stable in a 90:10 volumetric mixture of water to THF for 24 h (Figure S5). Size was monitored by DLS, and vials were visually scrutinized for aggregate formation. Representative photographs from visual inspection of various formulation outcomes are included as Figure S4.

## 4 Conclusions

The results presented here offer proof of principle for an extension of FNP technology that separates the nucleation and stabilization steps of nanoparticle assembly. Using two sequentially connected FNP mixers, nanoparticles can be formulated at higher core loadings and TMCs than in conventional single-step FNP. NPs with core loadings up to 90% were formulated without the aggregates that form via conventional FNP at the same mass loading and TMC. This improvement in loading and TMC are major advantages for this SNaP technique. Additionally, only minimal modification of existing FNP equipment is required to facilitate the sequential precipitation process.

When two CIJ mixers are used, the choice of amphiphilic stabilizer is limited to water-soluble species that can be introduced in an aqueous stream into the second mixer. Moving forward, we recommend that the second mixer be an MIVM for improved scalability and flexibility, as this would result in better in-mixer and downstream solvent quality control and would enable the use of a wider range of amphiphilic stabilizer chemistries, including those that are only soluble in organic solvents. A formulation prepared using this CIJ-to-MIVM setup using a vitamin E core and block copolymer PS-b-PEG stabilizer was developed and is reported in SI (Figure S3). However, manual operation of a CIJ-to-MIVM SNaP system is challenging and is limited by available syringe volumes and geometries. Independent control of stream flowrates (and thus solvent quality in the MIVM) can only be achieved using pumps to feed each stream into the mixers. Development of a lab setup for CIJ-to-MIVM and MIVM-to-MIVM operation is currently being pursued in our laboratory.

For formulations where NPs can be formed by both SNaP and conventional FNP, SNaP NPs are larger in diameter. We attribute some of this difference to NP assembly which occurs downstream of the final CIJ mixer in a less-controlled solvent environment. This would be solved by the CIJ-to-MIVM setup described above. However, it is conceivable that increasing the delay time between mixers could lead to tunable particle size in the 300–1000 nm range, which is currently difficult to achieve by FNP and which may have applications in depot delivery or microparticle formulation. This will be another focus of future studies.

**Funding sources**

Funding support has come from the Helen S Hunt Fund to the Princeton University School of Engineering and Science. We also acknowledge support from the Bill and Melinda Gates Foundation under grants INV-010674, INV-010674, and INV-042629.

## Declaration of Competing Interest

The authors declare that they have no known competing financial interests or personal relationships that could have appeared to influence the work reported in this paper.
