# Supplementary material for: Sequential Flash NanoPrecipitation for the scalable formulation of stable core-shell nanoparticles with core loadings up to 90%
Source: Int J Pharm. 2023 Jun 10;640:122985. doi: 10.1016/j.ijpharm.2023.122985 (PMC10262063; doi:10.1016/j.ijpharm.2023.122985)
Supplement: Supplementary data 1 [file mmc1.pdf]

Supplemental Information

Sequential Flash NanoPrecipitation for the scalable formulation of stable core-shell nanoparticles with core loadings up to 90%

Nicholas J. Caggiano[†,1], Satya K. Nayagam[†,1], Leon Z. Wang[1], Brian K. Wilson[1], Parker Lewis[1], Shadman Jahangir[1], Rodney D. Priestley[1,5], Robert K. Prud'homme[1,*], and Kurt D. Ristroph[*1,3]

[1]Department of Chemical and Biological Engineering, Princeton University, Princeton, New Jersey 08544, United States

[2]Princeton Materials Institute, Princeton University, Princeton, New Jersey 08544, United States

[3]Present address: Department of Agricultural and Biological Engineering, Purdue University, West Lafayette, Indiana 47909, United States

[*]Corresponding Author: ristroph@purdue.edu

[†]Contributed equally

Table S1. Stream compositions for conventional FNP control formulations studied in this work.

Formulation	CIJ #1-1 (Stream 1)	PCL Conc. (mg/mL)	Stream 1 Volume (mL)	CIJ #1-2 (Stream 2)	Stream 2 Volume (mL)	Reservoir Volume (mL)	Core Loading	TMC (mg/mL)	Size, t0 (nm)	PDI, t0
15	PCL in THF	5	0.5	HPMCAS in water + 15 mM NaCl	0.5	4	50%	1	157	0.16
16	PCL in THF	10	0.5	HPMCAS in water + 15 mM NaCl	0.5	4	50%	2	220	0.21
17	PCL in THF	20	0.5	HPMCAS in water + 15 mM NaCl	0.5	4	50%	4	agg.	agg.
18	PCL in THF	30	0.5	HPMCAS in water + 15 mM NaCl	0.5	4	50%	6	agg.	agg.
19	PCL in THF	6.25	0.5	HPMCAS in water + 15 mM NaCl	0.5	4	62.5%	1	196	0.15
20	PCL in THF	7.5	0.5	HPMCAS in water + 15 mM NaCl	0.5	4	75%	1	agg.	agg.
21	PCL in THF	8.25	0.5	HPMCAS in water + 15 mM NaCl	0.5	4	82.5%	1	agg.	agg.
22	PCL in THF	9	0.5	HPMCAS in water + 15 mM NaCl	0.5	4	90%	1	agg.	agg.
23	PCL in THF	9.5	0.5	HPMCAS in water + 15 mM NaCl	0.5	4	95%	1	agg.	agg.
24	PCL in THF	9.9	0.5	HPMCAS in water + 15 mM NaCl	0.5	4	99%	1	agg.	agg.
25	PCL in THF	5	0.5	DI water	0.5	4	100%	0.5	agg.	agg.
26	PCL in THF	5	0.5	15 mM NaCl in water	0.5	4	100%	0.5	agg.	agg.

Table S2. Stream compositions for SNaP formulations studied in this work.

Formulation	CIJ #1-1 (Stream 1)	PCL Conc. (mg/mL)	Stream 1 Volume (mL)	CIJ #1-2 (Stream 2)	Stream 2 Volume (mL)	CIJ #2-2 (Stream 4)	HPMCAS Conc. (mg/mL)	Stream 4 Volume (mL)	Reservoir Volume (mL)	Core Loading	TMC (mg/mL)	Size, t0 (nm)	PDI, t0
1	PCL in THF	5	1	15 mM NaCl in water	1	HPMCAS in water + 7.5 mM NaCl	2.5	2	6	50%	1	315	0.22
2	PCL in THF	10	1	15 mM NaCl in water	1	HPMCAS in water + 7.5 mM NaCl	5	2	6	50%	2	339	0.28
3	PCL in THF	20	1	15 mM NaCl in water	1	HPMCAS in water + 7.5 mM NaCl	10	2	6	50%	4	302	0.29
4	PCL in THF	30	1	15 mM NaCl in water	1	HPMCAS in water + 7.5 mM NaCl	15	2	6	50%	6	328	0.23
9	PCL in THF	6.25	1	15 mM NaCl in water	1	HPMCAS in water + 7.5 mM NaCl	1.875	2	6	62.5%	1	339	0.26
10	PCL in THF	7.5	1	15 mM NaCl in water	1	HPMCAS in water + 7.5 mM NaCl	1.25	2	6	75%	1	337	0.29
11	PCL in THF	8.25	1	15 mM NaCl in water	1	HPMCAS in water + 7.5 mM NaCl	0.875	2	6	82.5%	1	321	0.30
12	PCL in THF	9	1	15 mM NaCl in water	1	HPMCAS in water + 7.5 mM NaCl	0.5	2	6	90%	1	310	0.29
13	PCL in THF	9.5	1	15 mM NaCl in water	1	HPMCAS in water + 7.5 mM NaCl	0.25	2	6	95%	1	300	0.30
14	PCL in THF	9.9	1	15 mM NaCl in water	1	HPMCAS in water + 7.5 mM NaCl	0.05	2	6	99%	1	agg.	agg.

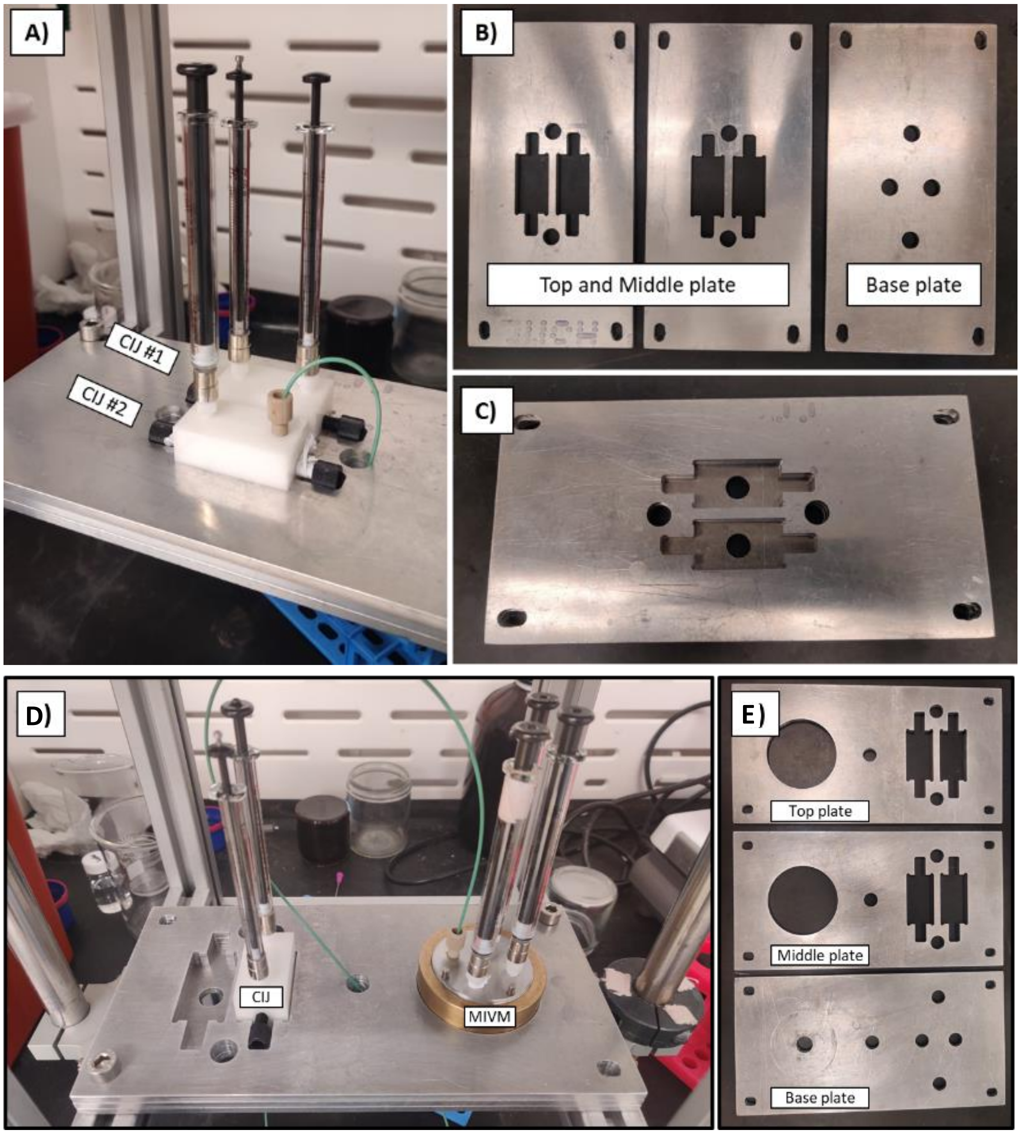

Figure S1. Stand mixing plate design and setup for sequential FNP process. **A)** Two connected confined impingement jet (CIJ) mixers used for sFNP. **B)** and **C)** Design of mixing stand plates to seat multiple CIJ mixers and allow for simultaneous depression of multiple syringes. **D)** Stand mixer setup for sequential FNP using a CIJ into an MIVM and **E)** base plate designs.

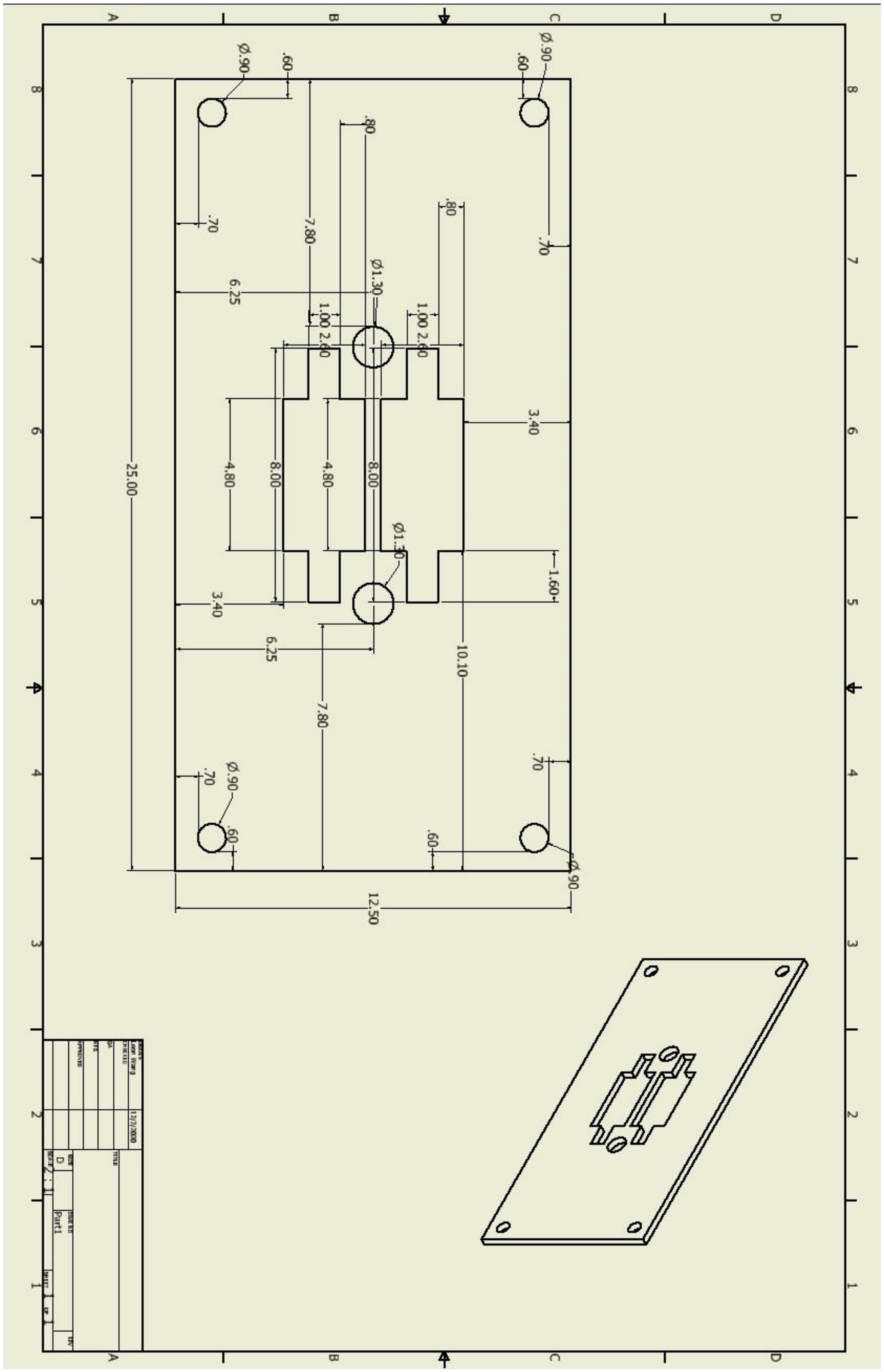

Figure S2-A. CAD drawing of top and middle plate for CIJ to CIJ setup.

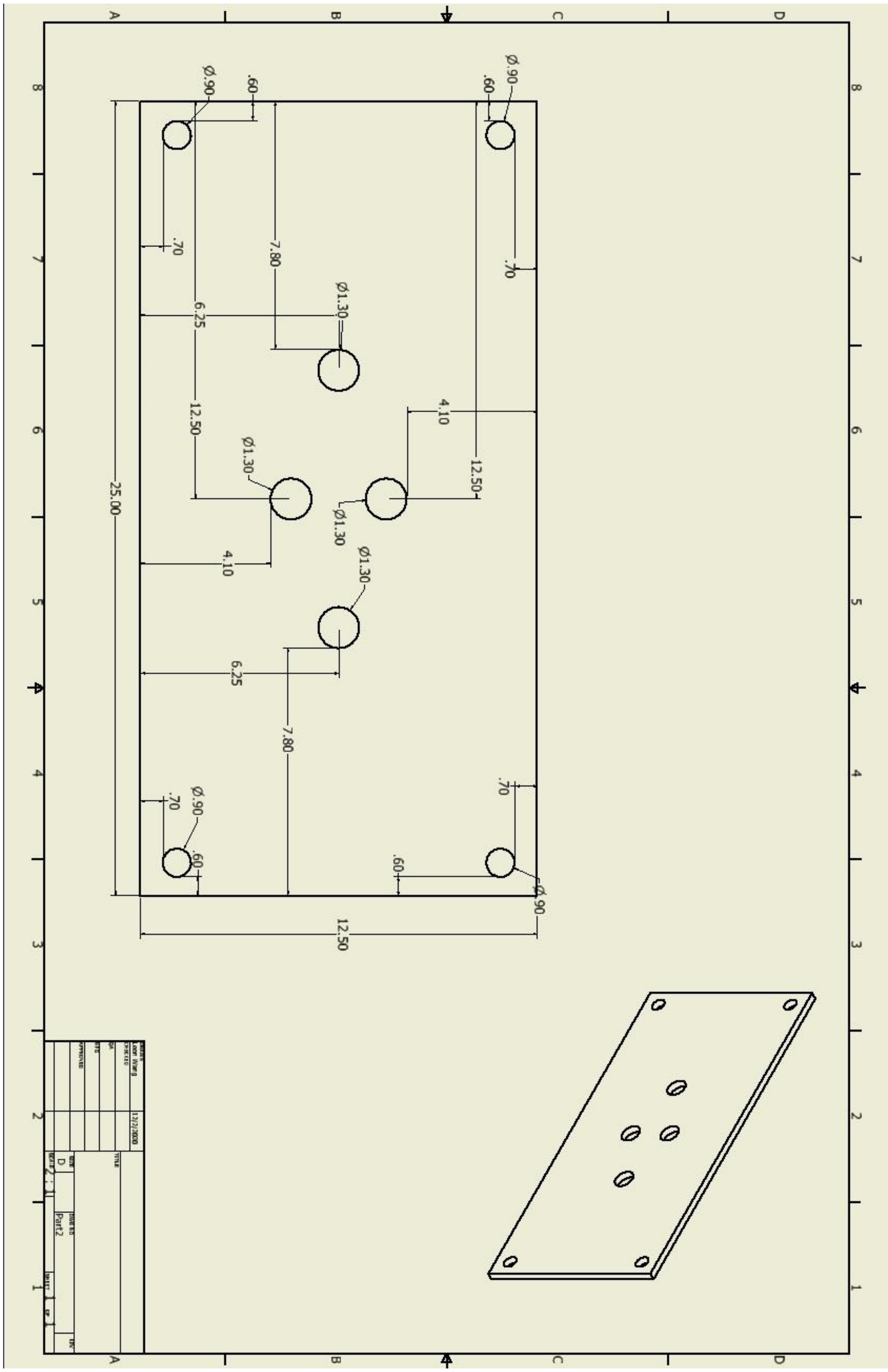

Figure S2-B. CAD drawing of bottom plate for CIJ to CIJ setup.

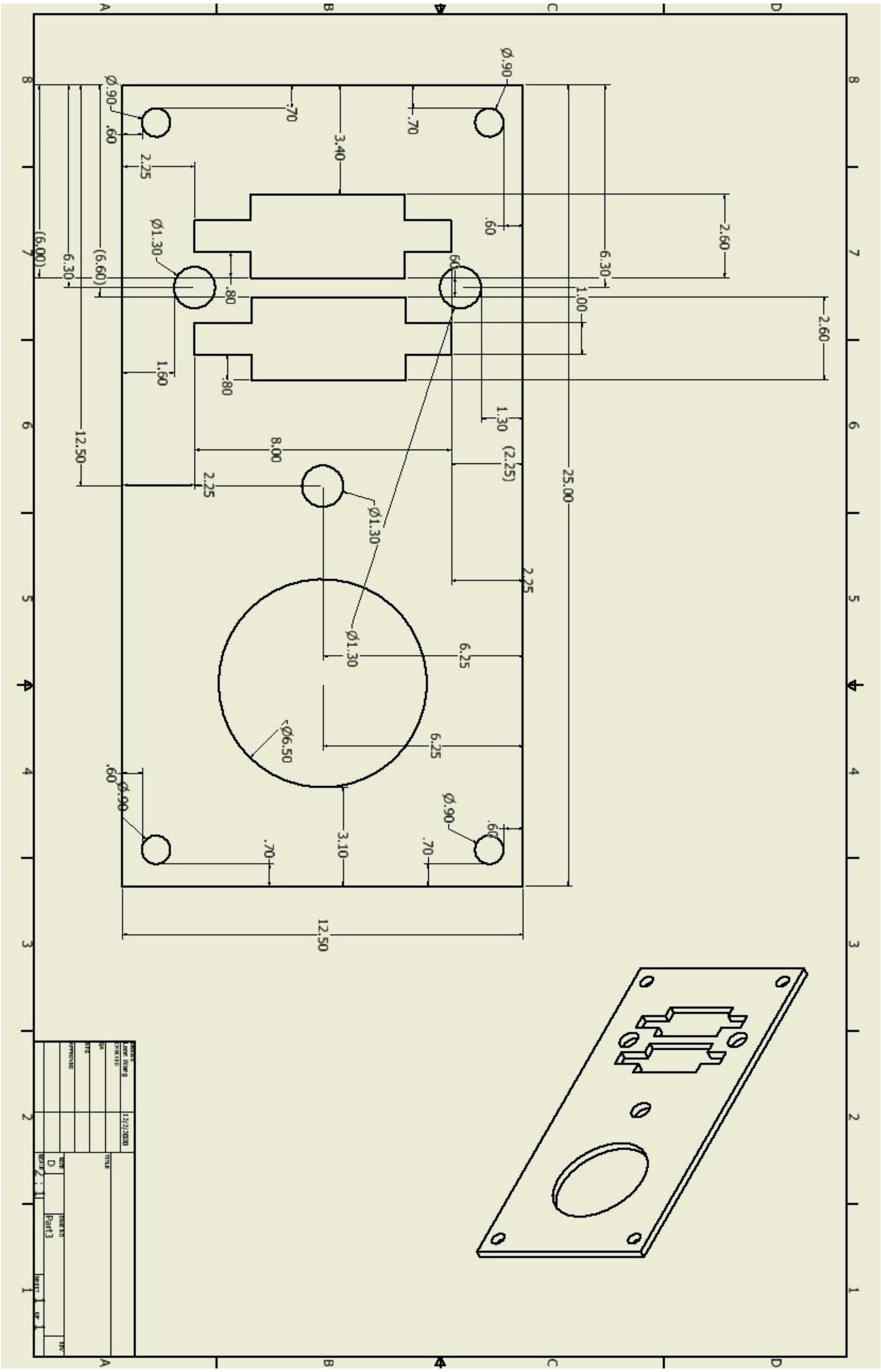

Figure S2-C. CAD drawing of top and middle plate for CIJ to MIVM setup.

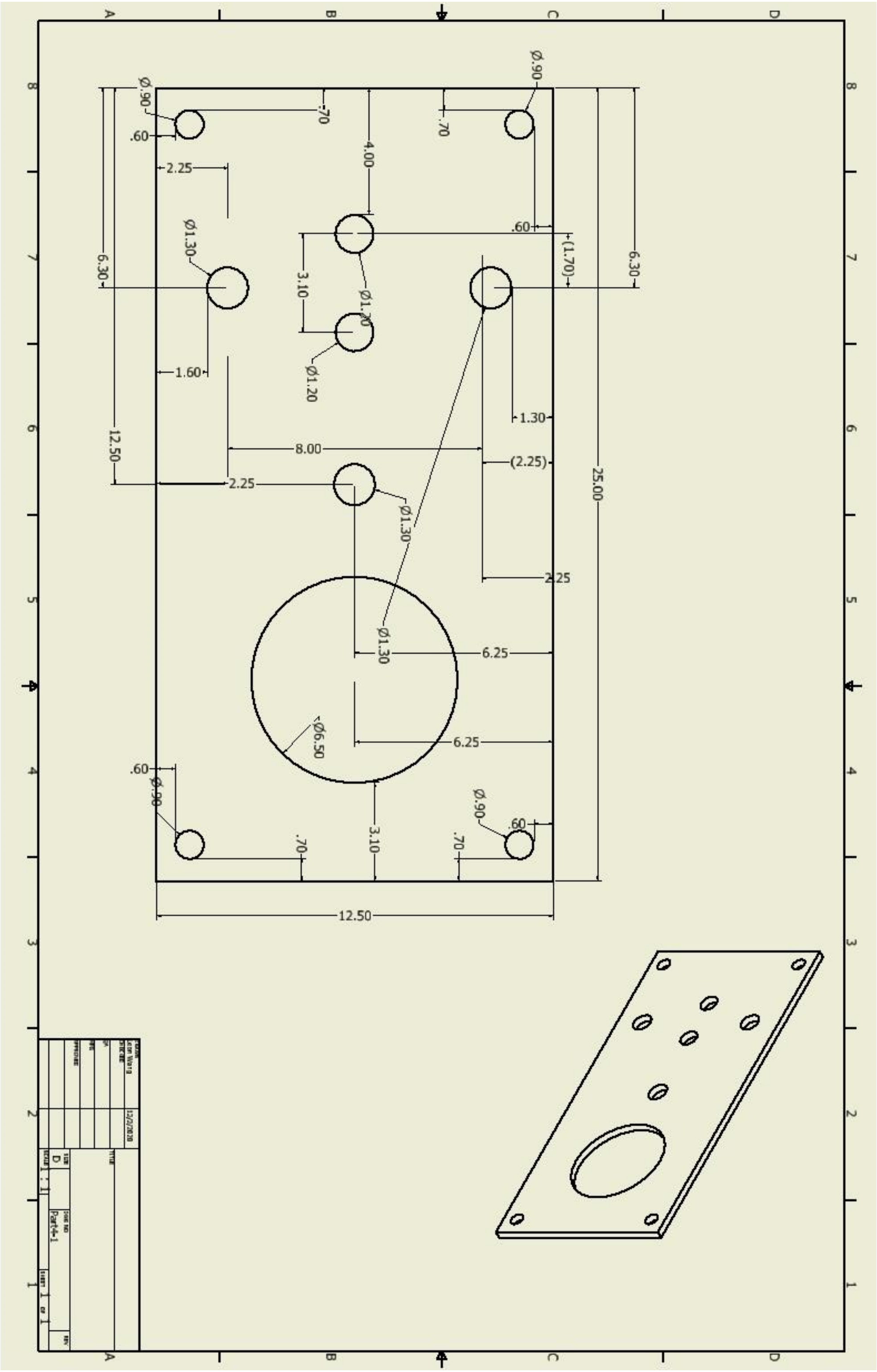

Figure S2-D. CAD drawing of bottom plate for CIJ to MIVM setup.

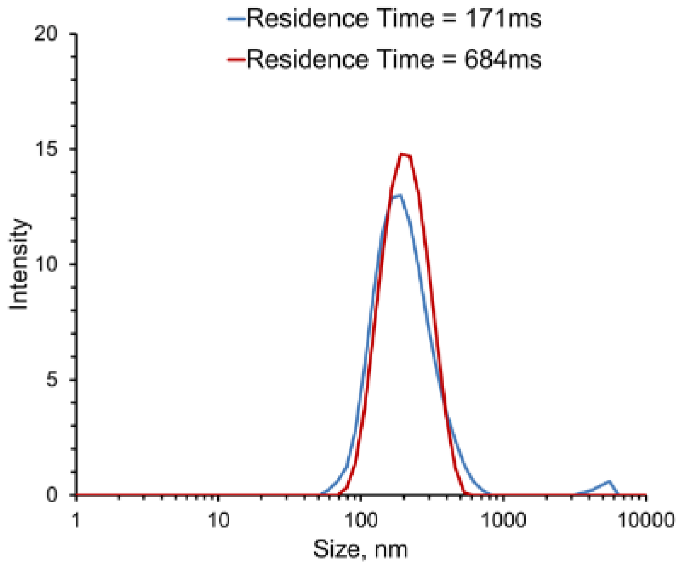

Figure S3. DLS trace for SNaP using a CIJ-to-MIVM setup to form NPs with a VitEAc core and PS-b-PEG shell at 83% core loading. Vitamin E acetate in THF was impinged against 0.1 M NaCl in water in the CIJ, and PS-b-PEG in THF was introduced in the MIVM along with two streams of 0.1 M NaCl in water. Varying the residence time between the two mixers from 171 ms to 684 ms using different length tubing did not appreciably affect size or PDI (181 nm, PDI = 0.19 and 189 nm, PDI = 0.18, respectively). PEEK tubing (ID = 0.75mm, OD = 1.58mm) was used to connect the outlet of one CIJ to the inlet of a multi-inlet vortex mixer (MIVM), using lengths of 210 mm or 840 mm to achieve the desired residence time.

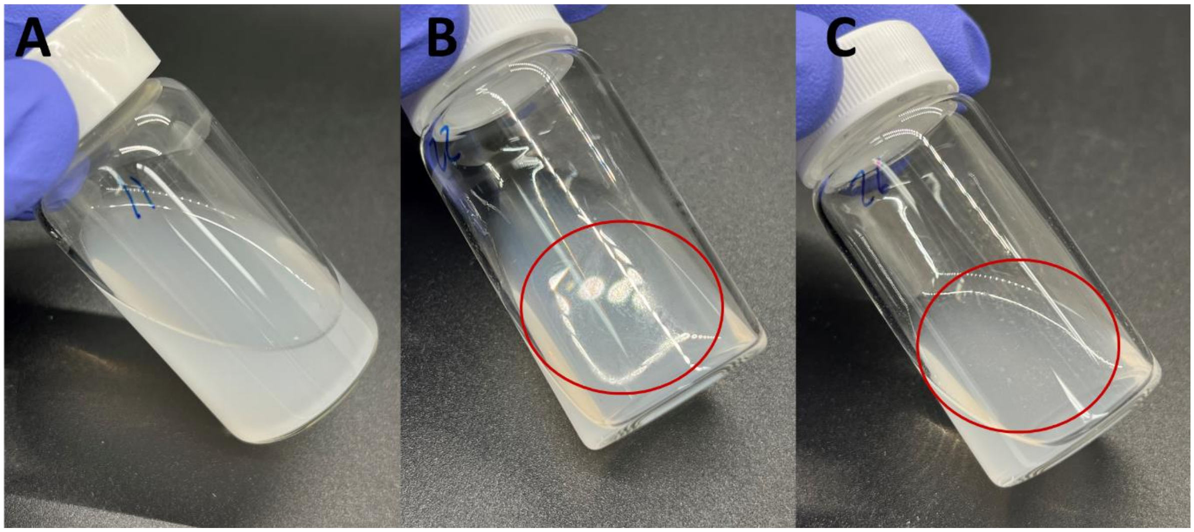

Figure S4. Representative photographs of NP suspensions. Aggregates are visible in panels B and C when vials are tilted and held under direct light. The formulation in vial A exhibited no aggregates.

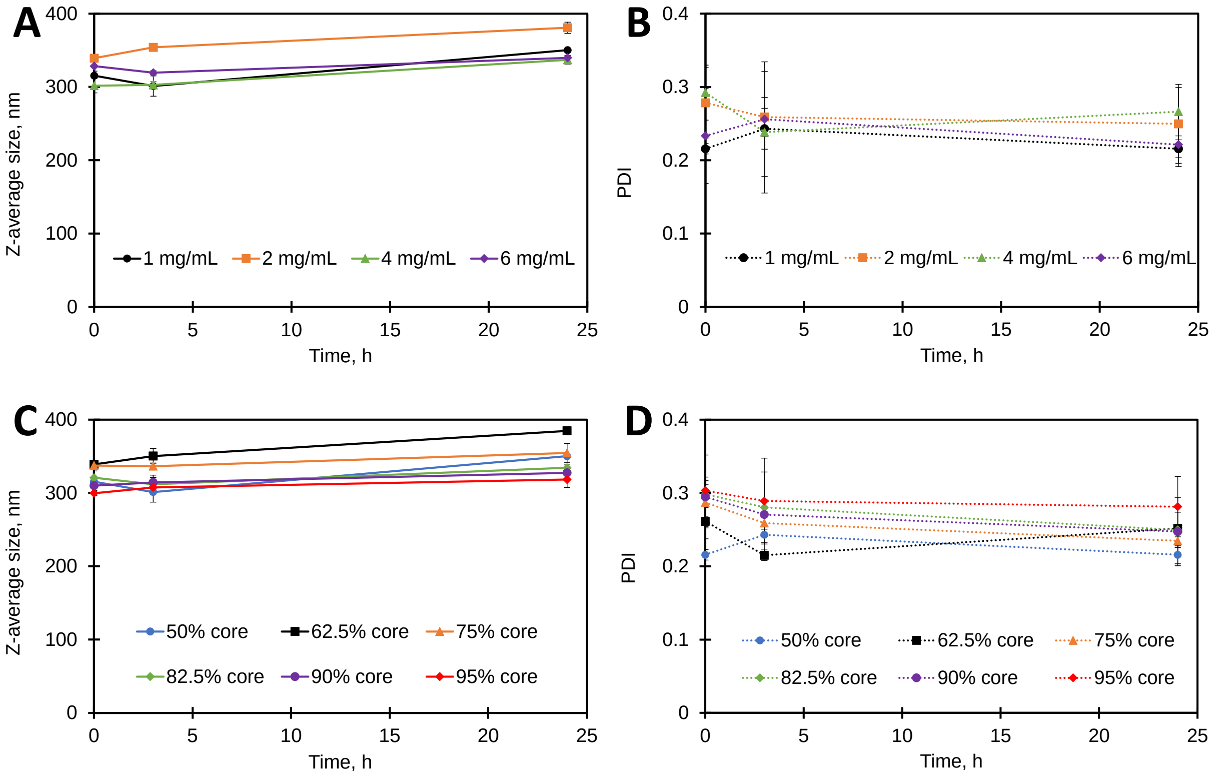

Figure S5. Size (A, C) and PDI (B, D) over time in 90/10 water/THF v/v for the SNaP formulations given in Figures 3 (A, C) and 4 (B, D) of the main text.
